# Ring-Opening Graft Polymerization of Propylene Carbonate onto Xylan in an Ionic Liquid

**DOI:** 10.3390/molecules20046033

**Published:** 2015-04-07

**Authors:** Xueqin Zhang, Mingjie Chen, Chuanfu Liu, Aiping Zhang, Runcang Sun

**Affiliations:** 1 State Key Laboratory of Pulp and Paper Engineering, South China University of Technology, Guangzhou 510640, China; E-Mails: xueqin0228@gmail.com (X.Z.); mangienew@gmail.com (M.C.); rcsun@scut.edu.cn (R.S.); 2 Institute of New Energy and New Material, Guangdong Key Laboratory for Innovative Development and Utilization of Forest Plant Germplasm, South China Agricultural University, Guangzhou 510642, China; E-Mail: aiping@scau.edu.cn; 3 Beijing Key Laboratory of Lignocellulosic Chemistry, Beijing Forestry University, Beijing 100083, China

**Keywords:** xylan, propylene carbonate, DBU, ring-opening graft polymerization, ionic liquid

## Abstract

The amidine organocatalyst 1,8-diazabicyclo[5.4.0]undec-7-ene (DBU) is an effective nucleophilic catalyst. Biocomposites with tuneable properties were successfully synthesized by ring-opening graft polymerization (ROGP) of propylene carbonate (PC) onto xylan using DBU as a catalyst in the ionic liquid (IL) 1-allyl-3-methylimidazolium chloride ([Amim]Cl). The effects of reaction temperature, reaction time and the molar ratio of PC to anhydroxylose units (AXU) in xylan were investigated. The physico-chemical properties of xylan-*graft*-poly(propylene carbonate) (xylan-*g*-PPC) copolymers were characterised by FT-IR, NMR, TGA/DTG, AFM and tensile analysis. The FT-IR and NMR results indicated the successful attachment of PPC onto xylan. TGA/DTG suggested the increased thermal stability of xylan after the attachment of PPC side chains. AFM analysis revealed details about the molecular aggregation of xylan-*g*-PPC films. The results also showed that with the increased DS of xylan-*g*-PPC copolymers, the tensile strength and Young’s modulus of the films decreased, while the elongation at break increased.

## 1. Introduction

The depletion of non-renewable energy and increasing environmental changes have stimulated human beings to tailor environmentally benign polymer replacements^___^biocomposites [1,2]. Biocomposites are the combination of a biodegradable polymer as the matrix material and biodegradable fillers (e.g., lignocellulosic) [2,3,4]. Over the past decades, lignocellulose has been recognized as the most important source for a broad variety of advanced polymeric materials for its excellent biodegradability, renewability, availability, acceptable mechanical and thermal properties compared to traditional carbon or aramid fibers [1,2,5,6]. The main components of lignocellulose are cellulose, hemicelluloses and lignin. However, cellulose and lignin have received more attention by far than hemicelluloses in terms of material application, although hemicelluloses represent about 20%–35% of lignocellulosic biomass [5,7,8].

Unlike cellulose which has a unique structure, hemicelluloses are heterogeneous polymers and the term is used to describe a group of polysaccharides composed of a combination of 5- and 6-carbon ring sugars. In the last decades, with the limitations of its heterostructure, research activities on hemicelluloses were aimed mainly at converting it into sugars, chemicals and fuel [9,10,11,12]. Stimulated by the shortage of natural energy sources, the structural variety and diversity of hemicelluloses has become more attractive, as they can be utilized in native or modified forms in biocomposites [9]. The interactions between natural fibers and the polymeric matrix play a key role in biocomposites’ properties [6]. However, the inherent drawbacks of hemicelluloses, such as poor solubility in common organic solvents, and low thermal stability during processing, lead to poor compatibility between the hydrophilic hemicellulosic fibers and the hydrophobic polymeric matrix [7,13]. To improve the compatibility between the fiber and polymer matrix, physical or chemical modifications of the fibers and/or polymer can be applied [7,14].

Among the various available modification methods, graft polymerization offers an attractive and versatile means of growing polymers directly off the fiber surface that depends more on which monomer and catalytic system that are utilised [15]. It is an efficient method to improve the properties of lignocellulose [16]. In order to achieve high operational efficiency, an effective monomer and an excellent reaction catalyst must be utilized. Aliphatic polyesters, including poly(ε-caprolactone) (PCL), poly(l-lactic acid) (PLLA), and poly(propylene carbonate) (PPC), which can be synthesized from cyclic monomers via ring-opening polymerization (ROP), resulting in controlled molecular weight and molecular weight distribution [7,17,18,19,20,21], have attracted increasing interest due to their functionality, biodegradability and biocompatibility [22,23]. These polymers are potential candidates as biocomposite matrixes. In many cases, several attempts have been conducted to create biodegradable composites using PCL and PLLA as polymeric matrixes to synthesis biocomposites derived from cellulose, chitosan and starch via graft polymerization [24,25,26,27]. PPC could be applied in tissue scaffolding, polymer electrolytes, adhesive agents and packaging materials [28]. The utilization of PPC materials can decrease the dependence on petroleum and reduce the massive emissions of carbon dioxide (CO_2_) [29]. However, research on PPC is mainly concentrated on it use in reinforced materials, such as PPC reinforced with unmodified granular cornstarch, PPC/starch-graft-poly (methyl acrylate) copolymer and PLLA/PPC blends [19,23,30]. As far as the authors are aware, there have been no reports on the graft polymerization of PPC with xylan.

In a heterogeneous catalyst system, propylene oxide and CO_2_ produce a regular alternating copolymer PPC, which shows high transparency and good biodegradability [23,31]. Since the first report of the use of 4-dimethylaminopyridine (DMAP) for the ROP of lactide in 2001 [32], several further reports have disclosed organocatalytic ROP using other nucleophilic catalysts, such as N-heterocylic carbenes (NHCs), 1,5,7-triazabicyclo[4.4.0]dec-5-ene (TBD) and 1,8-diazabicyclo[5.4.0]-undec-7-ene (DBU) [33,34,35]. Among them, DBU has proven to be a valuable catalyst and has been applied by a number of researchers [35,36,37]. However, no research has concentrated on graft polymerization of cyclic polyesters on a bio-matrix using DBU as catalyst. The aim of this study was to attempt grafting PPC with ring-opening graft polymerization (ROGP) of propylene carbonate (PC) onto xylan-type hemicelluloses using DBU as amidine organocatalyst in an ionic liquid (IL) 1-allyl-3-methylimidazolium chloride ([Amim]Cl). The effects of reaction temperature, reaction time and the molar ratio of PC to anhydroxylose units (AXU) in xylan were investigated.

## 2. Results and Discussion

### 2.1. Effects of Reaction Parameters on the Properties of Xylan-g-PPC Copolymers

In the present work, the xylan-*g*-PPC copolymers were tailored by varying the reaction conditions, including reaction temperature, reaction time and the molar ratio of AXU in xylan to PC (Table 1). The effects of reaction conditions on the degree of substitution (DS), the degree of polymerization (DP) and the weight percent gain (WPG) of xylan-*g*-PPC copolymers were examined. 

**Table 1:** Detailed structural factors of xylan-*g*-PPC copolymers obtained under different reaction conditions in [Amim]Cl.

Sample	Temp.	Time	AXU:PC	DS	DP	WPG ^a^ (%)
No	(°C)	(h)				
1	120	24	1:1	0.26	1.35	7.69
2	120	24	1:5	0.31	1.42	15.38
3	120	24	1:10	0.33	1.47	26.92
4	120	24	1:15	0.38	1.65	38.46
5	120	24	1:20	0.29	1.49	23.07
6	90	24	1:10	0.25	1.38	3.85
7	100	24	1:10	0.32	1.43	23.07
8	110	24	1:10	0.48	1.62	46.15
9	130	24	1:10	0.30	1.41	23.07
10	120	3	1:10	0.24	1.31	7.69
11	120	9	1:10	0.31	1.40	30.77
12	120	12	1:10	0.47	1.73	57.69
13	120	36	1:10	0.26	1.34	7.69

^a^ The weight percent gain of xylan due to the grafting of PPC side chains.

**Scheme 1:**
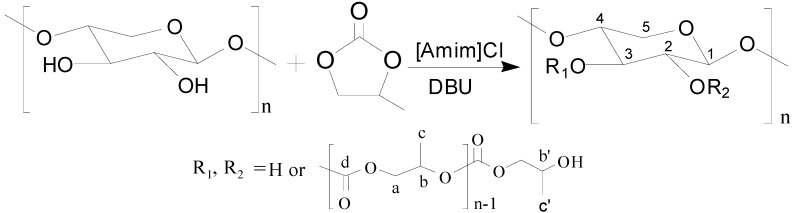
The ROGP of PC onto xylan in [Amim]Cl with DBU as catalyst.

Scheme 1 illustrates the ROGP reaction of PC with the hydroxyl groups on the backbone of xylan in [Amim]Cl with DUB as an amidine organocatalyst.

As shown in Table 1, with an increase of the molar ratio of AXU to PC from 1:1 to 1:15, the DS, DP and WPG of xylan-*g*-PPC copolymers were increased from 0.26 to 0.38, 1.35 to 1.65 and 7.69 to 38.46, respectively; while further increases of the molar ratio of AXU to PC from 1:15 to 1:20 led to the decrease in DS, DP and WPG. This decrease was probably due to the quick homopolymerization of PC compared with graft polymerization onto xylan under the high molar ratio. Keeping the molar ratio of AXU to PC at 1:10, the improvement of reaction temperature from 90 to 110 °C resulted in an increase in DS, DP and WPG from 0.25 to 0.48, 1.38 to 1.62 and 3.85 to 46.15, respectively, which was probably due to the diffusion and compatibility of the reaction ingredients and the mobility of the reactant molecules. Further increment of the temperature to 130 °C led to a slight decrease in DS, DP and WPG to 0.30, 1.41 and 23.07, respectively, which was probably due to the degradation of xylan at the high reaction temperature. Similarly, prolonging the reaction time from 3 h to 12 h, the DS, DP WPG were enhanced from 0.24 to 0.47, 1.31 to 1.73 and 7.69 to 57.69, respectively; while with further increases of the reaction time to 36 h, the DS, DP and WPG decreased to 0.26, 1.34 and 7.69, respectively, which may be due to the degradation of xylan and depolymerization of xylan-*g*-PPC copolymers in the ionic liquid at high temperature.

### 2.2. FT-IR Spectra

**Figure 1:**
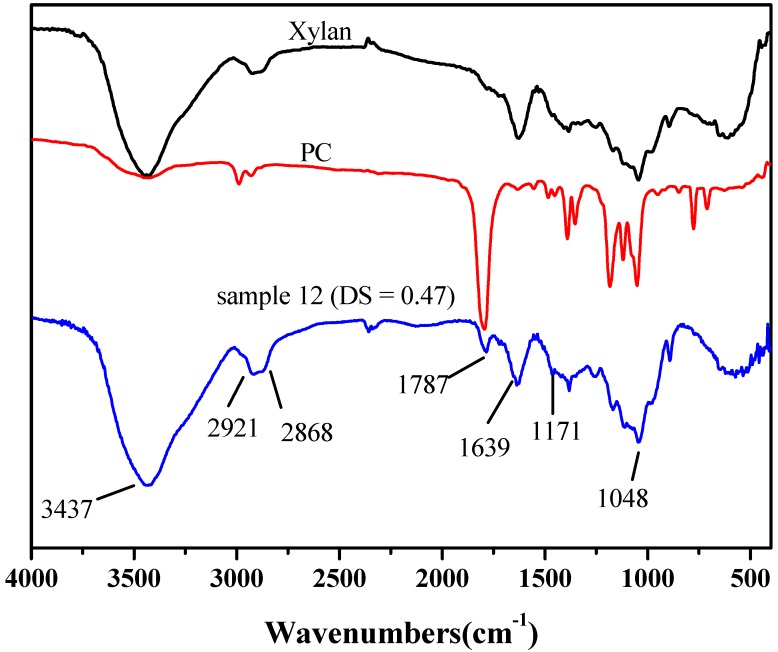
FT-IR spectra of unmodified xylan, pure PC and xylan-*g*-PPC copolymer sample 12 (DS = 0.47).

The FT-IR spectra of unmodified xylan, PC and xylan-*g*-PC copolymer sample 12 (DS = 0.47, DP = 1.73) are shown in Figure 1. The characteristic bands at 3437, 2868, 1639, 1048 and 891 cm^−1^ were previously reported [38,39]. Clearly, the spectrum of xylan-*g*-PPC copolymer provided direct evidence of the ROGP reaction by the changes of two important absorbances compared with that of unmodified xylan. The presence of the two new bands at 2921 cm^−1^ for CH_2_ stretching and 1787 cm^−1^ for C=O stretching indicated that new groups containing methylene and carbonyl moieties were attached onto xylan, suggesting the occurrence of the reaction illustrated in Scheme 1.

### 2.3. ^1^H-NMR, ^13^C-NMR and ^1^H-^13^C HSQC Spectra

**Figure 2:**
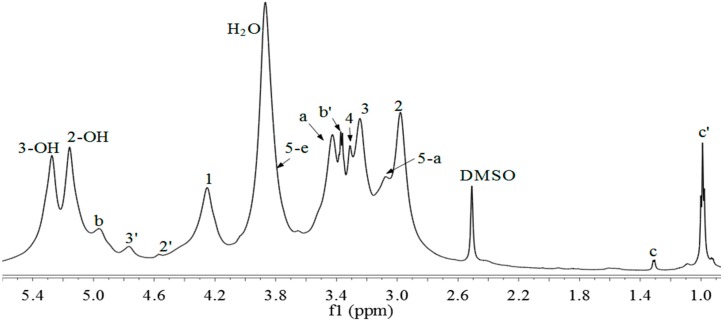
^1^H-NMR spectrum of xyaln-*g*-PPC copolymer sample 12 (DS = 0.47).

**Figure 3:**
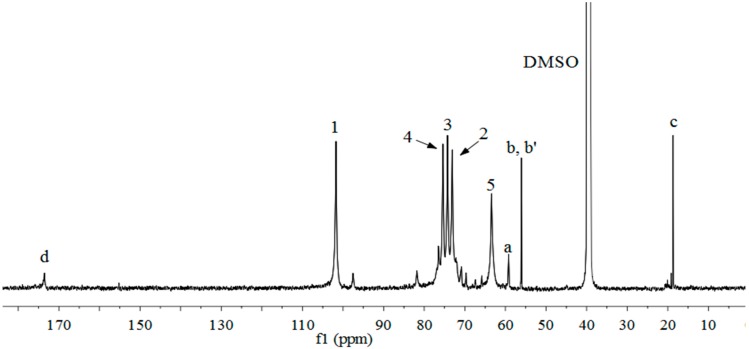
^13^C-NMR spectrum of xylan-*g*-PPC copolymer sample 12 (DS = 0.47).

**Figure 4:**
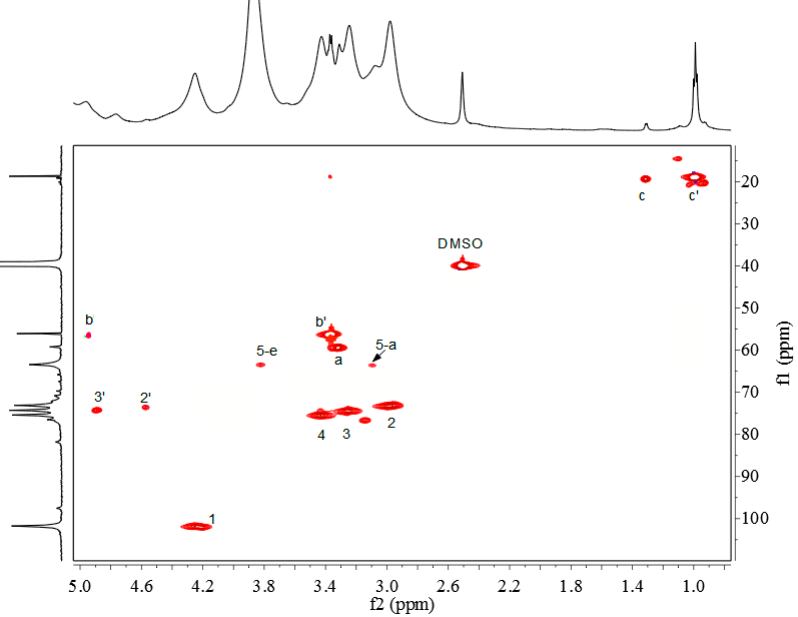
HSQC spectrum of xylan-*g*-PPC copolymer sample 12 (DS = 0.47).

The chemical structure of xylan-*g*-PPC copolymer was elucidated by ^1^H-NMR, ^13^C-NMR and HSQC (Figure 2, Figure 3 and Figure 4). As shown in Figure 2, the proton signals at 2.98, 3.08, 3.24, 3.31 and 4.25 ppm are assigned to H-2, H-5a, H-3, H-4, and H-1 of AXU in xylan, and those at 5.16 and 5.27 ppm are related to the protons from the hydroxyl groups in AXU [38]. The proton signal of H-5e is overlapped with the absorbed moisture (H_2_O) peak. The chemical shifts at 3.43, 4.96, 3.37, 1.31 and 0.99 are attributed to the protons at a, b, b', c and c' respectively. In addition, the signals at 4.57 and 4.77 ppm are assigned to the protons at substituted C-2 and C-3, respectively. These proton signals indicate the successful attachment of PPC side chains onto xylan backbone. According to the assignments, the DS and DP of the graft copolymers was estimated by the calculation with the peak intensity of corresponding signals based on the following equations (1) and (2):

(1) $$\text{DS}=\frac{{\text{PC}}_{\text{Terminal}}}{\text{AXU}}=\frac{{\text{I}}_{\text{b'}}}{{\text{I}}_{\text{H1}}}$$

(2) $$\text{DP}=\frac{{\text{PC}}_{\text{Total}}}{{\text{PC}}_{\text{Terminal}}}=\frac{{\text{I}}_{\text{b}}+{\text{I}}_{\text{b'}}}{{\text{I}}_{\text{b'}}}=\text{1}+\frac{{\text{I}}_{\text{b}}}{{\text{I}}_{\text{b'}}}$$

where DS is the degree of substitution of PPC, DP is the degree of polymerization of PPC, AXU is anhydroxylose unit, PC_Terminal_ is the end unit of PPC, PC_Total_ is the total units of PPC, I_b_ and I_b'_ are the integral area of the resonances of the methylene protons at b and b', and I_H1_ is the integral area of the resonance assigned to H-1 of AXU.

The DS and DP values estimated from ^1^H-NMR are listed in Table 1. The results indicated that the xylan derivatives with DS 0.24–0.47 and DP 1.31–1.73 were obtained under the selected conditions.

The ^13^C-NMR spectrum of xylan-*g*-PPC copolymer sample 12 is shown in Figure 3. The main five signals at 101.8, 73.1, 74.3, 75.3 and 63.4 ppm are assigned to C-1, C-2, C-3, C-4 and C-5 of AXU in xylan, respectively [39,40]. The signals at 59.1, 56.1, and 18.6 ppm correspond to the a, b and b', and c positions of side chain in PPC, respectively. The signal at 173.5 ppm is assigned to the carbonyl at the d position, indicating the successful attachment of PPC onto the xylan backbone.

To confirm the correct assignment of the main signals of the xylan-*g*-PPC copolymers, the ^1^H-^13^C HSQC spectrum was collected, as illustrated in Figure 4. HSQC is a powerful tool for quantitative and qualitative analyses of chemical structures. Strong correlations for AXU were clearly observed, which were previously reported [27]. As expected, the strong correlations at δ_H_/δ_C_ of 3.33/59.3, 4.95/59.1, 3.36/55.73, 1.31/19.4 and 0.99/19.4 were attributed to a, b, b', c and c', respectively. The presence of these strong correlations indicated the successful graft polymerization reaction of PPC onto xylan. In addition, the two correlations at δ_H_/δ_C_ of 4.58/72.6 and 4.88/74.3 are assigned to substituted C-2 and C-3, indicating the attachment of PPC occurred at C-2 and C-3 positions of AXU. Based on the quantitative integrations of substituted and unsubstituted C-2 and C-3, the results indicated that 59.31% and 40.69% of PPC side chains were attached to the C-3 and C-2 positions of AXU, respectively.

### 2.4. Thermal Analysis

**Figure 5:**
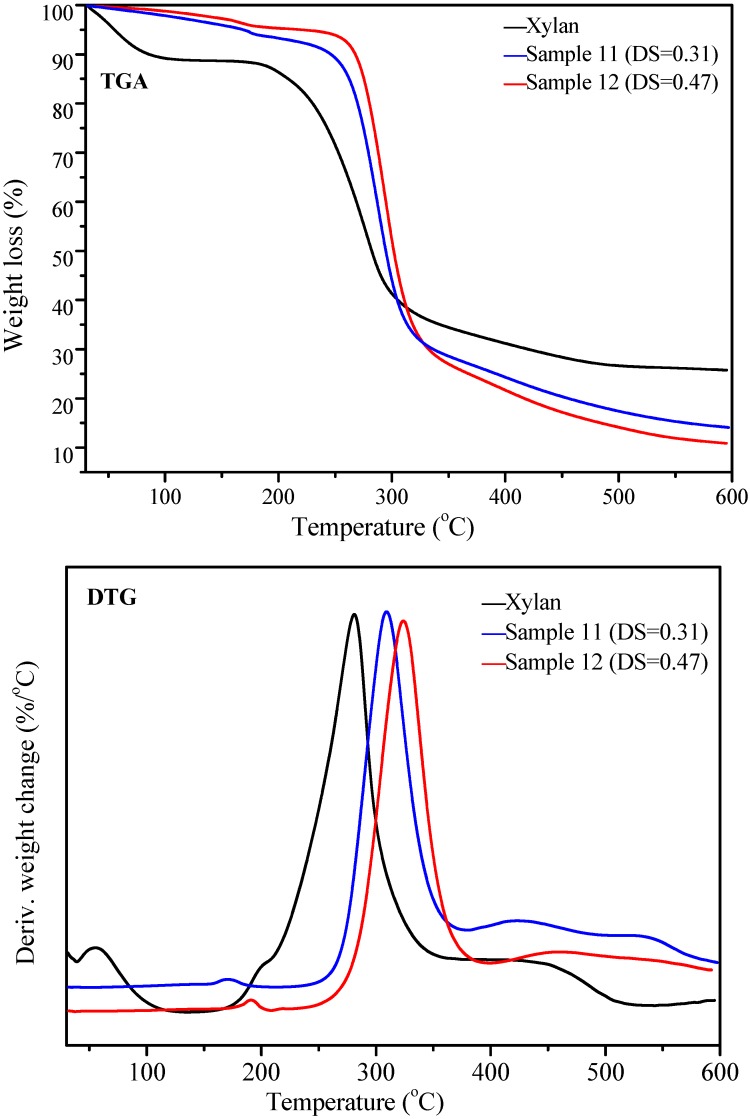
TGA/DTG curves of unmodified xylan and xylan-*g*-PPC copolymers sample 11 (DS = 0.31) and sample 12 (DS = 0.47).

The thermal stability of unmodified xylan and xylan-*g*-PPC copolymers sample 11 (DS = 0.31, DP = 1.40) and sample 12 (DS = 0.47, DP = 1.73) were investigated using themogravimetric analysis (TGA) and derivative thermogravimetry (DTG) in the temperature range from 30 to 600 °C under nitrogen atmosphere, and the corresponding TGA/DTG curves are shown in Figure 5. In TGA curves, the weight loss can be divided into three stages: minor weight loss below 150 °C, substantial weight loss and the subsequent marginal weight loss at high temperature. At the first stage, a sudden drop in the curve of unmodified xylan below 150 °C is due to the lost water, which represents about 11% of the initial weight of xylan. In the case of xylan-*g*-PPC copolymers, minimum weight loss was observed, representing about 5% and 4% for sample 11 and sample 12, respectively, suggesting the increased hydrophobic nature after ROGP reaction [5]. At the second stage, the substantial weight loss was due to the decomposition of samples. Unmodified xylan began to decompose at 195 °C, while xylan-*g*-PPC copolymers samples 11 and 12 started to decompose at 252 °C and 269 °C, respectively, indicating the increased thermal stability after the attachment of PPC. At 50% weight loss, the decomposition temperature occurred at about 285 °C, 290 °C and 300 °C for unmodified xylan and xylan-*g*-PPC copolymers samples 11 and 12, respectively, implying the increased thermal stability of xylan after ROGP reaction. At the last stage, the pyrolysis residues at 600 °C were 25% for xylan, higher than 15% and 10% for sample 11 and sample 12, respectively.

To further explore the decomposition process of xylan and xylan-*g*-PPC copolymers, DTG curves were also recorded and are displayed in Figure 5. DTG represents the degradation rate and can be used for the comparison of the thermal stability between the samples. The primary peak in DTG curves showed the maximum degradation rate at 280 °C for unmodified xylan, 310 °C for sample 11, and 330 °C for sample 12, indicating that the attachment of PPC side chains onto xylan led to the increased thermal stability of xylan.

### 2.5. Effect of DS and DP on the Mechanical Properties of Xylan-g-PPC Copolymer Films

**Figure 6:**
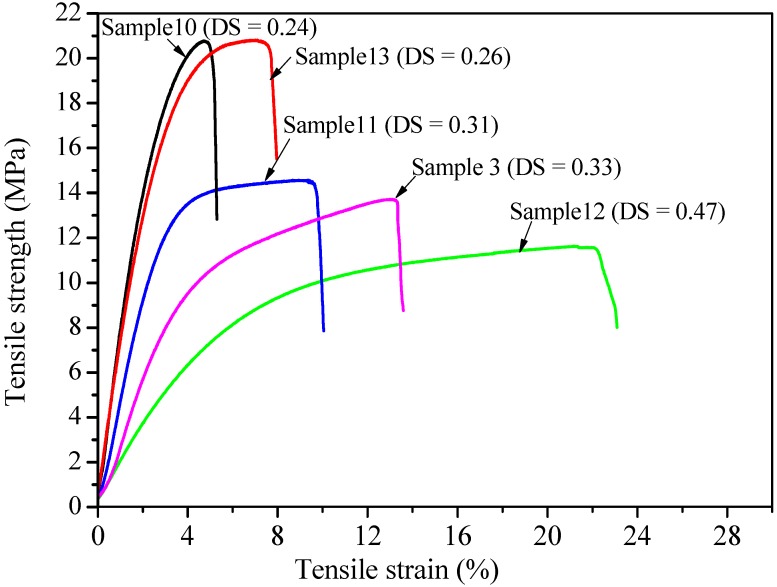
Tensile strain curves of xylan-*g*-PPC copolymers films.

Tensile strength and tensile strain at break are important mechanical properties of xylan-*g*-PPC copolymer films, mainly depending on the DS and DP of xylan-g-PPC copolymers. The tensile strength, Young’s modulus and tensile strain at breaks of films are shown in Table 2, and the typical stress-strain curves are shown in Figure 6. It is known that unmodified xylan hardly form films, whereas xylan-*g*-PPC copolymer sample 10 with a DS of 0.24 and DP of 1.31 could easily form films with a tensile strength of 20.78 MPa, Young’s modulus of 658 MPa and a moderate tensile strain (4.75%). The improved mechanical performance for the xylan-*g*-PPC copolymers are obviously attributable to the good compatibility between xylan and PPC after graft polymerization. However, further increasing DS and DP of xylan-*g*-PPC copolymers, both the tensile strength and the Young’s modulus of the films decreased, while the tensile strain increased. As shown in Figure 6, the highest tensile train is about 22.13%. 

**Table 2:** Mechanical properties of the films produced from xylan-*g*-PPC copolymers.

Sample (S)	DS	Tensile Strength (MPa)	Tensile Strain at Break (%)	Young’s Modulus (MPa)
10	0.23	20.78 ± 3.2	4.75 ± 1.1	658 ± 38
13	0.24	20.76 ± 2.9	7.73 ± 2.5	632 ± 36
11	0.30	14.48 ± 2.8	9.79 ± 2.7	343 ± 31
3	0.32	13.11 ± 2.3	13.23 ± 3.7	329 ± 29
12	0.45	11.32 ± 2.1	22.13 ± 3.8	126 ± 22

Pure PPC exhibits rubber-like characters including low elastic modulus, low tensile strength and high elongation at break [29]. Comparatively, the grafting of PPC onto xylan could enhance the properties of PPC-based composites. With the increase of DS and DP, the tensile strain correspondingly increased, and the tensile strength and the Young’s modulus of films decreased due to the increased content of PPC in xylan-g-PPC copolymers.

### 2.6. Morphology of Xylan-g-PPC Copolymer Film Surface

**Figure 7:**
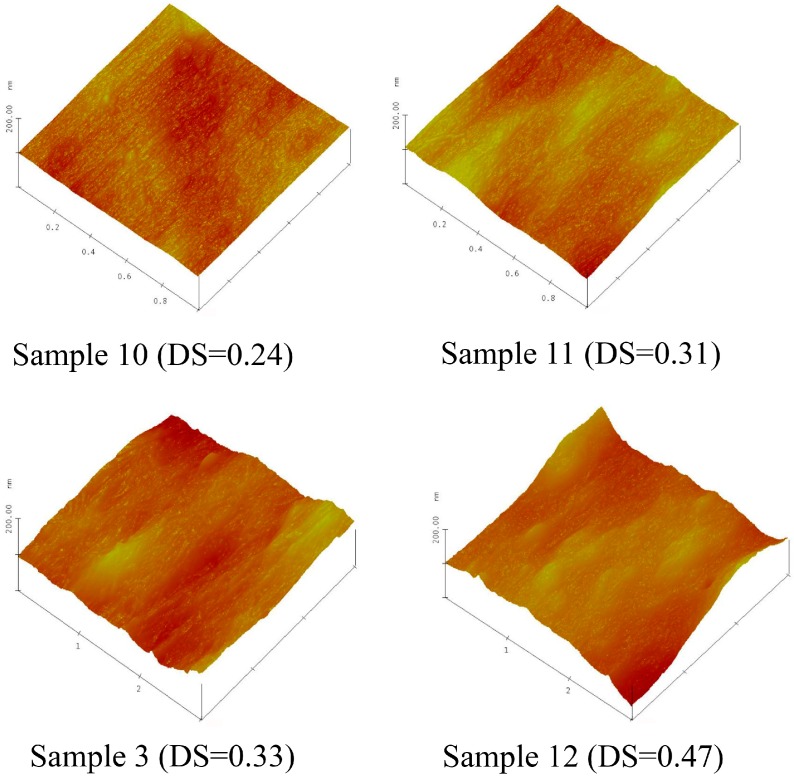
AFM images of xylan-*g*-PPC copolymers.

Atomic Force Microscopy (AFM) images were also recorded to determine the surface structure of the films, and the representative images are illustrated in Figure 7. By observing the surface morphologies of the films with different DS and DP, a large difference in the structure of the films was visualized. Xylan-*g*-PPC film with a low DS of 0.24 and DP of 1.31 displayed uniform surface. When DS increased to 0.31 and DP to 1.40, the film surface became relatively rough and showed some nodules. Further increasing DS and DP from 0.33 to 0.47 and 1.47 to 1.73, nodules became more apparent, which was probably due to the attachment of PPC onto the xylan backbone resulting in the molecular aggregation of PPC side chains on the surface of xylan-*g*-PPC copolymers films. With the development of DP, the molecular aggregation of PPC side chains intensified. 

## 3. Experimental Section

### 3.1. Materials

Xylan with xylose content of over 85% was purchased from Yuan-Ye Biological Technology Co., Ltd. (Shanghai, China). [Amim]Cl with purity of 99% was supplied by Cheng-Jie Chemical Co., Ltd. (Shanghai, China), and dried in vacuum for 48 h at 70 °C before use. Propylene carbonate (PC) and DBU with a purity of 99% were purchased from Aladdin Reagent Co. (Shanghai, China). All other chemicals were of analytical reagent grade and directly used without further purification. 

### 3.2. Homogeneous Preparation of Xylan-g-PPC Copolymers with Different Reaction Parameters

The preparation of xylan-*g*-PPC copolymers was performed in [Amim]Cl under the conditions in Table 1 according to the following procedure: dry xylan (0.33 g, 0.005 moles of hydroxyl group in xylan) was added to [Amim]Cl (10 g) in a dried 50 mL three-neck flask. The mixture was agitated with magnetic stirring at 80 °C for about 2 h under the protection of nitrogen to achieve a homogeneous solution. Then PC was introduced into the xylan solution at the required temperature. The ROGP reaction was carried out under the protection of nitrogen with magnetic stirring for 24 h. After the precipitation in 150 mL ethanol, the solid residues were filtered out and thoroughly washed with ethanol. The products were suspended in dichloromethane with magnetic agitation at room temperature for 24 h (thrice, 72 h total) to remove PPC homopolymers. The solid residues were filtered out, dried in vacuum at 60 °C for 48 h and stored away from moisture. The products were weighed to determine the yield of ROGP reaction, expressed by WPG, on the basis of the oven-dried measurements according the following equation:

(3) $$\text{WPG}=\frac{{\text{m}}_{2}-{\text{m}}_{1}}{{\text{m}}_{1}}\times \text{100\%}$$

where WPG is the weight percent gain after ROGP reaction, m_1_ is the weight of xylan regenerated in IL, and m_2_ is the weight of xylan-g-PPC copolymers after the treatment of dichloromethane.

### 3.3. Characterization

FT-IR spectra of unmodified xylan and xylan-*g*-PPC copolymers were recorded on a Tensor 27 spectrophotometer (Bruker, Karlsruhe, Germany) from a KBr disc containing 1% (w/w) finely ground samples in the range 4000–400 cm^−1^.

The ^1^H-NMR, ^13^C-NMR spectra of unmodified xylan and xylan-*g*-PPC copolymers were recorded from 40 mg samples in 0.5 mL DMSO-*d*_6_ on an Avance III 600 M spectrometer (Bruker) with a 5 mm multinuclear probe. For ^1^H-NMR analysis, the detailed collecting and processing parameters were as follows: number of scans, 16; receiver gain, 31; acquisition time, 2.7263 s; relaxation delay, 1.0 s; pulse width, 11.0 s; spectrometer frequency, 600.17 MHz; and spectral width, 12019.2 Hz. For ^13^C-NMR analysis, the detailed collecting and processing parameters were as follows: number of scans, 2770; receiver gain, 187; acquisition time, 0.9088 s; relaxation delay, 2.0 s; pulse width, 12.0 s; spectrometer frequency, 150.91 MHz; and spectral width, 36057.7 Hz. The DS and DP of xylan-*g*-PPC copolymers were calculated from the integration of the resonances assigned to characteristic signals in ^1^H-NMR spectra.

The thermal stability of the samples was performed by TGA and DTG using a Q500 theromgravimetric analyzer (TA Instruments, New Castle, PA, USA). The apparatus was continually flushed with nitrogen. The sample between 9 and 11 mg was heated from 30 °C to 600 °C at a heating rate of 10 °C/min. 

The surface morphology was determined by AFM analysis (Nanoscope III, Veeco Co. Ltd., Plainview, NY, USA). AFM scanning was conducted at four locations on each sample. Topographic (height) and phase images were recorded in tapping mode under ambient air.

### 3.4. Film Preparation

The synthesized xylan-*g*-PPC copolymers (0.25 g) were dissolved in DMSO (5 mL) at room temperature with magnetic stirring. Then the solution was cast into a polystyrene dish to form wet films. The films were dried in an oven at 50 °C for 6 h and stored in plastic bags for characterization. The tensile strength of xylan-*g*-PPC copolymer films was determined from rectangular specimens (15 mm × 10 mm) on a Universal Testing Machine 5565 (Instron, Norwood, MA, USA) fitted with a 100 N load cell at 23 °C with 50% RH.

## 4. Conclusions

In this study, xylan-*g*-PPC copolymers were successfully synthesized by ROGP reaction of PC onto xylan in [Amim]Cl with DBU as an amidine organocatalyst. The DS and DP of xylan-*g*-PPC copolymers can be controlled by varying the reaction parameters, including reaction temperature, the molar ratio of AXU to PC, and reaction time. FT-IR and NMR analyses indicated that PC was successfully grafted onto the xylan backbone. After the attachment of PPC side chains, the thermal stability of the copolymers was higher than that of unmodified xylan. Considering the good tensile strength of the copolymer films and the good biodegradability of xylan and PC, these xylan derivatives have great potential application as food packaging materials.
